# Building resilience for future adversity: a systematic review of interventions in non-clinical samples of adults

**DOI:** 10.1186/s12888-014-0227-6

**Published:** 2014-08-14

**Authors:** Tania Macedo, Livia Wilheim, Raquel Gonçalves, Evandro Silva Freire Coutinho, Liliane Vilete, Ivan Figueira, Paula Ventura

**Affiliations:** Institute of Psychology, Federal University of Rio de Janeiro, Rio de Janeiro, Brazil; Department of Epidemiology, National School of Public Health (ENSP-FIOCRUZ), Rio de Janeiro, Brazil; Institute of Psychiatry, Federal University of Rio de Janeiro, Rio de Janeiro, RJ Brazil

**Keywords:** Resilience, Prevention, Health promotion

## Abstract

**Background:**

Potentially traumatic events happen in people’s lives, leading to the risk of the development of posttraumatic stress disorder, depression and even suicide. Resilience is an individual’s ability to maintain or regain his/her mental health in the face of significant adversity or risk of death. The aim of this study was to conduct a systematic review of studies evaluating the effectiveness of resilience promotion interventions in adults.

**Methods:**

Electronic searches were conducted in databases ISI, PsycINFO and PubMed, including every language and every year until January 20, 2013. We selected studies with nonclinical samples of adults that evaluated the effectiveness of the intervention through randomized and non-randomized controlled trials and open-ended studies. We also considered valid constructs directly related to resilience, such as hardiness.

**Results:**

Among 2.337 studies, 13 were selected for the review, 5 through electronic databases and 8 through search in references or the “times cited list” (list of articles that cited the selected papers). Of these, 7 are randomized controlled trials, 5 non-randomized controlled trials, and one an open-ended trial. Most of the studies included reported some degree of improvement in resilience-like variables among those subjects exposed to resilience-promoting programs. Furthermore, positive findings were more consistent among randomized controlled trials - six out of the seven suggested efficacy.

**Conclusion:**

There is evidence pointing towards some degree of effectiveness of resilience promotion programs, despite the poor operationalization of the construct and great heterogeneity in the studies. Indeed, the analysis of the methodological quality of the selected studies was hampered by the poor quality of reporting. There were faults in reporting in most studies on almost all items (random sequence generation, allocation concealment, blinding of outcome assessment, incomplete outcome data, description of concurrent treatment and intent-to-treat analysis), except for the item “selective reporting”. Additional efforts should be made to determine the actual effect size of the interventions, since this is crucial for calculating the cost-effectiveness of resilience promotion strategies.

**Electronic supplementary material:**

The online version of this article (doi:10.1186/s12888-014-0227-6) contains supplementary material, which is available to authorized users.

## Background

“Bad things happen” [1], as Bonnano et al. put it. Nobody escapes the adversities of life, whether they are potentially traumatic events, such as the sudden death of a loved one, or setbacks in personal or work life. Traumatic events happen frequently [2,3]. Numerous epidemiological studies have shown high lifetime rates of prevalence, for example 90% in the US [4], 76% in Mexico [5], 64.6% of men and 49.5% of women in Australia [6], 81% of men and 74% of women in Canada [7]. Most people are exposed throughout their lives to at least one event capable of eliciting an emotional response of such high magnitude as to be characterized as “psychological trauma”, according to the DSM –IV [8]. Despite the high prevalence of traumatic events, there is an evident variability in the adaptation to such events and a natural heterogeneity of the human stress response. Posttraumatic Stress Disorder (PTSD) affects 5-10% of individuals exposed to these incidents. According to Bonanno et al. [1,9] among the possible outcomes after exposure to traumatic events, resilience is the most commonly observed response pattern. When the exposure is more prolonged or severe, when it involves interpersonal violence or occurs during childhood, the prevalence of PTSD or other mental disorders can reach higher levels, but it hardly ever exceeds 30% of the sample [10,11].

There is an array of possible ways of defining the key constructs related to resilience, such as positive adaptation to adversities/stress and hardiness. Therefore, studying such a complex construct is a challenge. From the perspective of trauma researchers, resilience is defined as the effective adaptation after significant threats to personal and physical integrity [12]. Hoge et al. [13] characterize resilience as not developing PTSD after a trauma. Bonanno et al. [14] conceptualize resilience as the absence of PTSD symptoms or the presence of a single symptom of PTSD. Masten [15] highlights that the individual needs to receive a significant threat, such as severe adversity or an exposure to a traumatic event, in order for resilience to take place. Moreover, she believes that the quality of adaptation needs to be good. Luthar & Zigler [16] call attention to how best to define a positive adaptation to adversity. Initial studies investigated competence based on external criteria of adaptation, such as social, academic and behavioral competence, expected for a given age and a given culture [16]. Luthar & Zigler [16] argue that internalizing and externalizing symptoms need to be assessed, because although individuals may show competence in behavioural indices, they may have a variety of other psychological difficulties. Masten [15] also suggests that narrowing the definition of positive adaptation only to the absence of PTSD can lead to erroneously classifying people as resilient who may in fact suffer from a disorder other than PTSD [17,18].

Despite the complexity of the construct, several scales have been developed to measure “resilience”, and they actually appear to measure individual, social or family characteristics which can facilitate the process of resilience. In order to assess resilience in children and adolescents, some early studies used scales examining specific resilient characteristics and risk factors, such as locus of control [16]. Several studies have used scales to examine competence or coping skills, two qualities that may be related to resilience [13]. In a methodological review of resilience measurement scales [19], all of the measures had missing information regarding the psychometric properties. The scales that received the best ratings were the Connor-Davidson Resilience Scale, the Resilience Scale for Adults and the Brief Resilience Scale. Hoge et al. [13] argue whether resilience can be measured by a set of questions at a single point in time, as opposed to observing the subject during a stressful experience and then determining how well the person returns to normal functioning. In this scenario, resilience scales would measure an individual’s reaction to an experimental paradigm or stressful life event overtime. Hoge et al. [13] also indicate that resilience scales should be used and observed in a trauma-exposed control group without PTSD, because these would probably be the individuals most likely to be truly resilient. However, studies assessing resilience in such groups are not available.

The promotion of resilience has potential implications for the prevention of mental disorders in professionals who are constantly exposed to risks, such as rescue workers and members of armed forces. Rescue workers, for instance, have high exposure to traumatic events and the pooled current worldwide prevalence of PTSD in this group of professionals is 10% [20]. The military is another group deserving greater attention regarding prevention strategies in order to reduce the health, social and economic burdens of mental disorders. The National Vietnam Veterans Readjustment Study (NVVRS) reported a life-time prevalence of warzone-related PTSD of 30.9% among the 3.1 million men and women who served during the Vietnam War [21]. Hoge et al. [22] assessed 2,530 men and women in US combat infantry units, before deployment to Iraq, and 3,671 after return from Iraq and Afghanistan. Rates of PTSD, major depression and generalized anxiety disorder ranged from 15.6 to 17.1% after deployment in Iraq and 11.2% after deployment in Afghanistan. Suicide rates have increased exponentially among American soldiers since the beginning of the wars in Iraq and Afghanistan [23]. The number of suicides committed after mission in Afghanistan has already exceeded the total number of U.S. military personnel who have died in combat in the same country [24]. To deal with this scenario, increasing research targeting secondary and tertiary prevention as well as treatment of mental disorders has been undertaken. However, there is a dearth of investigations targeting primary, pretrauma prevention of mental disorders in health samples in risk professions. Therefore, the development of evidence-based and research-informed resilience-building interventions have been considered essential to the success of primary mental disorders prevention programs [25].

Indeed, the great variability of the stress responses suggests the hypothesis that protective factors may prevent, weaken or attenuate the effects of stress, avoiding the development of mental disorders [26,27]. The existence of these protective factors increases the possibility of developing strategies for preventing psychiatric disorders and reducing the negative effects of adversity on individuals’ mental health, i.e., the possibility of promoting resilience. Following this line of prevention, there are several studies being published [28-40]. However, syntheses of the current knowledge about the efficacy or inefficacy of resilience-promoting programs are highly relevant, as these programs are already being widely implemented on a large scale. For example, the US Army, aiming at preventing or reducing the adverse psychological effects of combat, is submitting more than 900,000 soldiers and veterans to the Comprehensive Soldier Fitness (CSF) program with a cost of approximately 125 million dollars [41]. The CSF is based on the Penn Resiliency Program (PRP), typically a school-based program designed for youths in late childhood and early adolescence, although it has been evaluated in other settings, including primary care clinics and juvenile detention centers [42]. This massive training and research program has been criticized for being released without pilot testing to assess the effectiveness of such training in a military environment [43].

To the best of our knowledge, there are no systematic reviews or meta-analysis studies focusing on non-clinical adult samples. Thus, the purpose of this article is to systematically review studies that evaluate the effectiveness of resilience promotion in adults, where the intervention has focused on encouraging emotional resilience (or related constructs), in order to strengthen the individual against future stressors or adverse situations.

## Method

Electronic searches were performed in the ISI, PsycINFO and PubMed databases, including every language and every year until January 20, 2013. The terms used in the individual search were the following:

ISI (advanced search):

- TS = (“behavi* therapy” OR “cognitive therapy” OR “cognitive behavio* therapy” OR CBT OR “cognitive reest*” OR “positive psychology” OR “well being therapy” OR “anxiety manage*” OR relaxation OR “stress control training” OR “stress inoculation training” OR “stress inoculation” OR “progressive relaxation” OR “diaphragmatic breathing” OR “social abilit* training” OR “social skills training” OR psychotherap*)
- TS = (“resilienc*” OR “Subjective well-being” OR “positive psychology” OR “health promotion” OR “cognitive flexibility” OR “post-traumatic growth” OR “stress-related growth” OR hardiness)
- TS = (protocol OR program OR treatment OR promotion)

All databases were activated and the survey included only “articles” and “notes”. The results of each individual search were combined with “and”.

In PsycINFO/PsychLit, we searched directly in “Any Field” and limited the search by including only “Journal Article” and excluding “Chapter”, “Dissertation” or “Book Review”. The same search terms were used in the ISI database. As in the ISI, the results of each individual search were combined.

In PubMed, we performed the search in “All Fields”, and the groups of terms used and combined between each other were as follows:

- (“cognitive therapy” OR CBT OR “positive psychology” OR relaxation OR “stress inoculation training” OR “stress inoculation” OR “progressive relaxation” OR “diaphragmatic breathing” OR “social skills training” OR psychotherap*)
- (resilience OR resiliency OR “positive psychology” OR “health promotion” OR “cognitive flexibility” OR “post-traumatic growth” OR “stress-related growth” OR “hardiness”)
- (protocol OR program OR treatment OR promotion)

In addition to searches in online databases, manual searches were performed in the reference list of selected articles and times cited lists (ISI database).

The criteria for including the studies in the review were: 1) evaluation of efficacy through studies with randomized controlled designs, non-randomized controlled trials, or open-ended trials of resilience training programs; 2) the purpose of the training program was to strengthen resilience, in order to prepare the individual to cope with future adverse events. Constructs directly related to resilience, such as hardiness or stress inoculation, were also considered valid; 3) the studies were conducted with non-clinical adult samples.

The exclusion criteria used for considering the studies for the review were: 1) studies which were not primarily designed to promote resilience, such as those with a focus on increased well-being or positive emotions; 2) theoretical articles or reviews, book chapters, theses or dissertations; 3) studies which focused on children or adolescents; 4) research that focused on strengthening resilience in physically and/or mentally ill individuals or with a primary focus on assessing resilience in the aftermath of exposure to specific traumatic events (e.g. resilience training after natural disaster); 5) case reports or case series (not open trial); for this instance, we used the definition of Pincus et al. (1993) stating that open trials should include 10 cases or more; 6) studies without a standardized efficacy measure before and after the intervention; 7) studies which performed baseline evaluation without intervention; 8) studies in which the resilience concept was related to another area of study (e.g., physics or mathematics); 9) studies which evaluated the effects of physical activity or yoga, rather than psychological programs to promote resilience; 10) animal studies.

After the search phase, we performed an analysis of the methodological quality of each article selected for this review, based on an adapted version of the Cochrane Collaboration Tool for Assessing the Risk of Bias [44]. This tool suggests items through which it is possible to assess the risk of bias in each study. We selected seven items that would represent risk of bias consistent with randomized studies (random sequence generation, allocation concealment, blinding of outcome assessment, incomplete outcome data, selective reporting, description of concurrent treatment and intent-to-treat analysis) and four risk items consistent with non-randomized studies (blinding of outcome assessment, incomplete outcome data, selective reporting and description of concurrent treatment).

## Results

**Figure 1:**
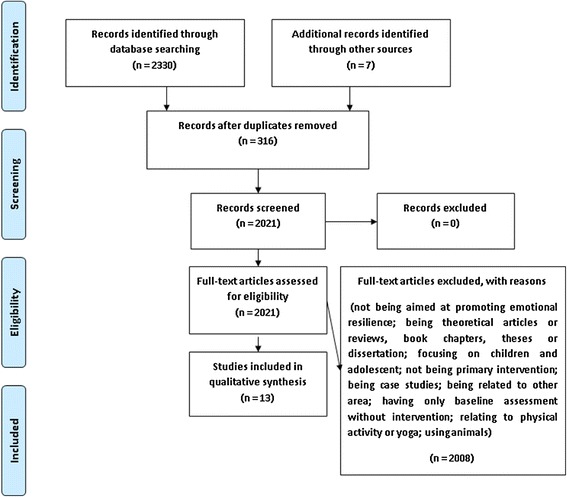
**Flowchart of the process of identifying and selecting studies.**

The results of our search can be seen in the flowchart (Figure 1).

Thus, a final set of 13 studies [28-40] were selected for this review (5 of these studies through electronic databases and 8 of them through searches in references or times cited lists). Out of these, 7 were randomized controlled trials, 5 were non-randomized controlled trials, and 1 was an open-ended trial. An additional table file shows each of the selected studies [see Additional file 1].

Of the seven randomized controlled trials considered for this review, three used “resilience scales,” one measured the hardiness construct, and three used, as resilience surrogates, scales that assess process and factors related to this construct (such as coping, self-esteem, locus of control, social support and positive affect). The three studies which measured resilience through validated construct-measuring scales found statistically significant change, proving the effectiveness of the intervention. Of the three studies which used as resilience surrogates scales that assess process and factors, two of them found significant changes in the administered scales. The study which assessed hardiness obtained a significantly more positive result. Thus, six out of the seven randomized controlled trials suggested efficacy.

Among the five non-randomized controlled studies, one of them used the concept of hardiness, and four of them used the concept of resilience. The study which measured hardiness found significant change in relation to the control group after the intervention. Among the four studies which investigated resilience, only one controlled study employed a “resilience scale”, showing a significant increase of resilience. The other three studies used as resilience surrogates scales that assess process and factors related to this construct and found significant changes regarding only some of the factors. Therefore, the non-randomized studies were less consistent than the randomized ones with regard to the efficacy of the intervention.

The samples investigated were composed of employees of companies [29,35], bank employees [38], employees of a university [40], industrial workers [32], customer service professionals [36], managers [28,33], students [31,37], soldiers [30,39] and physicians [34].

The intervention programs used different approaches (positive psychology techniques, CBT, transformational coping, acceptance and commitment therapy, mindfulness, interpersonal therapy, attention and interpretation therapy, relaxation and diaphragmatic breathing).

As for the format of the interventions, four studies used online interventions [32,33,35,36], eight studies used group interventions, and in one study the intervention was based on face-to-face therapy [34]. The number and duration of the sessions varied as well. The shortest intervention had a total of 80 minutes [30] while the longest lasted hours [29]. Some studies did not specify the exact duration of their interventions [38,39]. Since there are only a few studies in total, it is not possible to determine which techniques, approaches, formats, and durations of intervention are most effective in promoting resilience.

Only three of the seven randomized controlled trials had follow-up data (10 weeks [29,33] and 23 days [30]). Among the five non-randomized controlled studies, three had follow-up data (6 months [35,36,39]).

### Assessment of methodological quality

**Figure 2:**
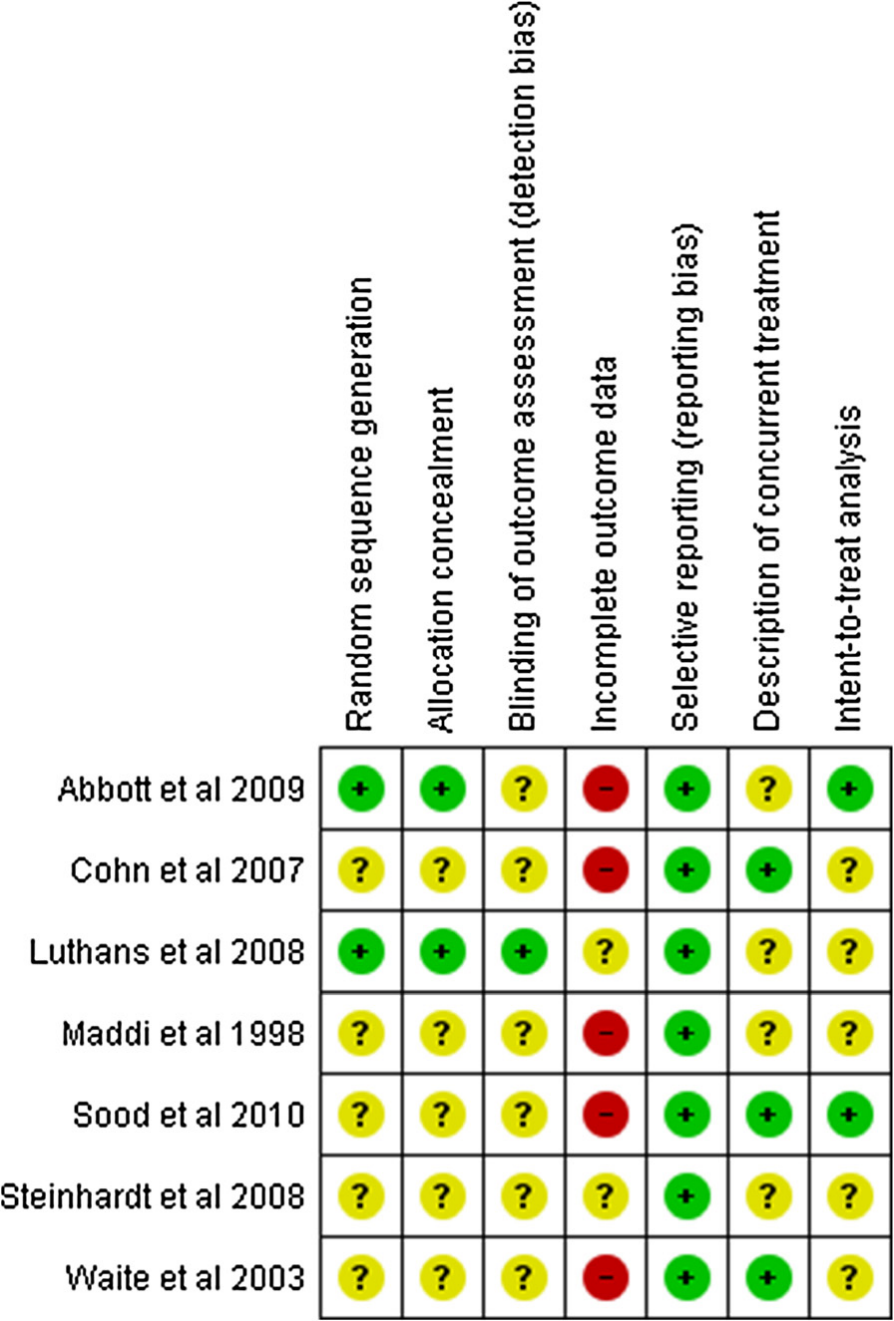
**Methodological analysis of randomized controlled trials; + low risk of bias; - high risk of bias; ? Unclear risk of bias.**

**Figure 3:**
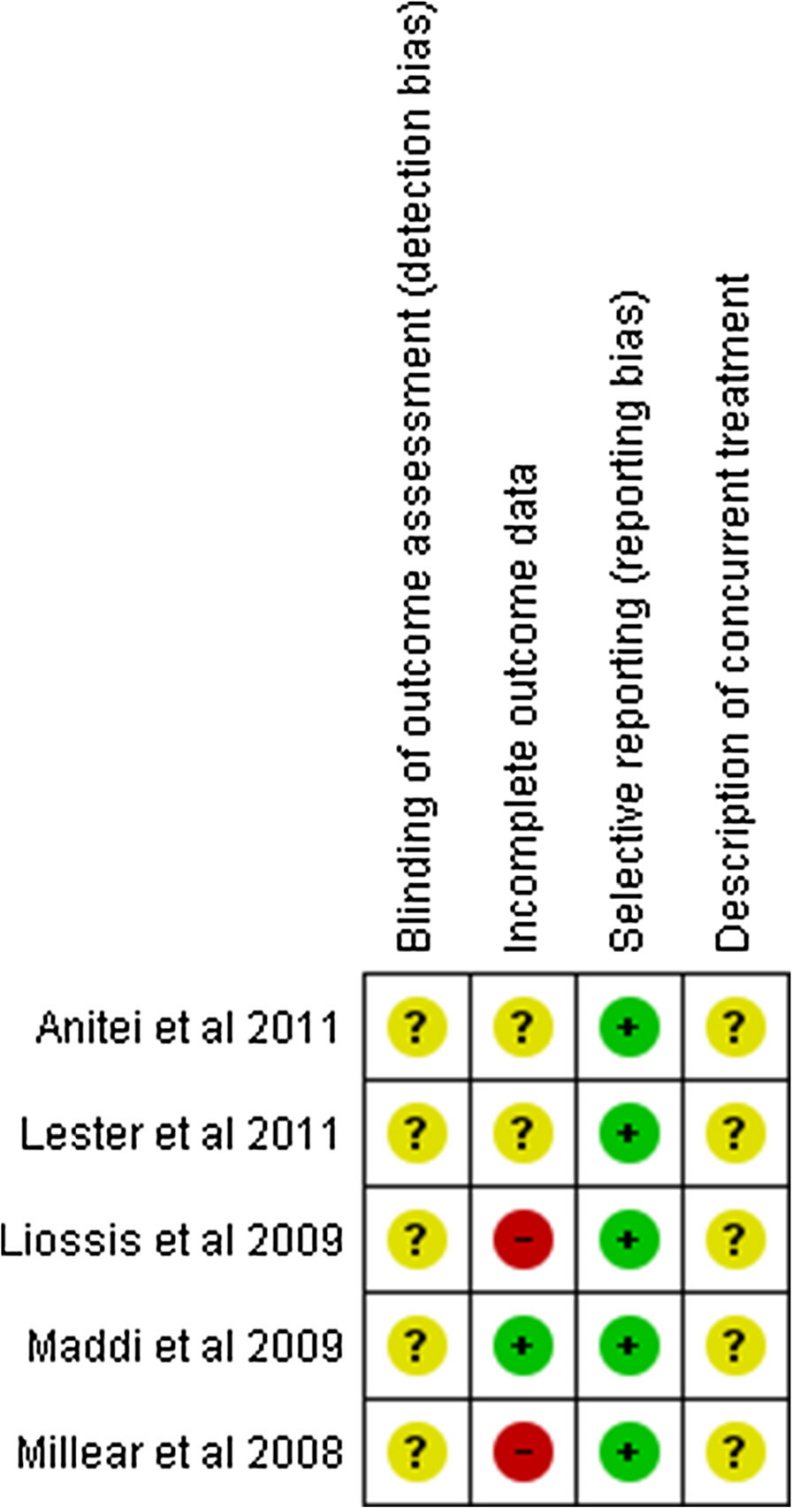
**Methodological analysis of non-randomized controlled trials; + low risk of bias; - high risk of bias; ? Unclear risk of bias.**

Figures 2 and 3 summarize the different aspects concerning the methodological quality of the randomized and non-randomized studies. Only two out of the seven randomized studies provided information about the random sequence generation and allocation concealment, and thereby ensuring that there was no selection bias in the participants for each group. Only one study reported the existence of blinding of outcome assessment. Regarding incomplete outcome data, none of the randomized studies included complete data about all subjects (including the number of dropouts) in the final analysis, as two of them did not provide any information at all about data loss, and five had a high dropout rate. All of the studies provided data from all of the questionnaires administered at the beginning and end of the research, not omitting possible negative results. Three studies prevented participants from being subjected to other psychotherapeutic treatment at the time of the study, while four did not provide this data. Finally, only two studies performed intention-to-treat analysis.

None of the five non-randomized studies reported whether the assessors were blind to the condition of the participant, only one of these studies included data from all participants in the final analysis, while two of them did not provide this piece of information and did not include data from all participants. Finally, no study reported whether the subjects were undergoing other psychotherapeutic interventions at the same time.

## Discussion

This systematic review is aimed at investigating the effectiveness of resilience-promoting programs, which are designed to strengthen individuals, in order for them to cope with future adversity. As far as we know, there has only been one previous systematic review/meta-analysis summarizing the evidence for the effectiveness of these programs. Brunwasser et al. [42] performed a meta-analysis to assess the effectiveness of the PRP, which showed no evidence of it being superior to active control conditions. The program had modest and inconsistent effects [43]. One major difference between the Brunwasser et al. [42] study and our research is that the first focused more on seeking to determine whether PRP was effective in targeting depressive symptoms, whereas our study attempted to assess the effectiveness of promoting resilience in order to cope with adversity in general. In addition, there is a great difference between the samples of the two studies: Brunwasser et al. [42] focused on children and adolescents, while this systematic review focused on nonclinical adult samples.

### Limitations of the studies

The selected studies had several limitations. The main one is concerning the lack of standardization of the resilience concept across the different studies included in this review. While this is common in emerging fields, unification of the concept is needed, so that the evaluation of the intervention effectiveness can be more objective. This is expressed, for example, in the variety of scales used by the authors.

As noted, few studies reported on follow-up data. Since adverse situations are random and unpredictable, it is important that resilience acquired after the intervention is maintained long term. However, we cannot say that the individual who received the intervention will be able to use the acquired skills if adversity occurs in the medium to long term.

Furthermore, no study has investigated the occurrence of adverse situations after intervention. That is, in all of the studies, the improvement in resilience was detected through a change in scores in “resilience scales” or of resilience-related factors. According to Masten & Coatsworth [45], for resilience to be identified, there needs to be a significant threat to the individual and, when facing such threat, the quality of adaptation or development needs to be good. Individuals who have never suffered significant threat cannot be considered resilient. Therefore, we cannot conclude that individuals who showed increased scale scores after the intervention will be effectively resilient after a traumatic event.

Furthermore, the selected studies assessed only the potential benefits of the interventions. No study has considered the possibility of the intervention posing some risk to the participants, nor reported data of adverse effects or the possibility of worsening after preventive action. Bonanno et al. [1] warned, for example, about suicide and eating disorder prevention programs that had negative effects on a portion of participants.

### Assessment of methodological quality

Analysis of the methodological quality of the selected studies was greatly hampered by the poor reporting of the studies. Among the randomized clinical trials, information on five of the seven quality items was missing in more than half the studies. It is important to point out that the large majority of these trials were published after the CONSORT Statement of 2001, an expert consensus that listed the items deemed fundamental to reporting standards for a randomized clinical trial, had appeared in three very prestigious medical journals [46,47].

Concerning the included non-randomized studies, none of them provided information on blinding of outcome assessment and description of current treatment. It is also important to stress that all the included non-randomized papers were published after the STROBE statement^53^ appeared in 2007 in several medical journals. The Strobe represents for observational studies what Consort is for randomized controlled trials.

So, for the reasons stated above, the evaluation of the methodological quality of the individual studies was compromised by the large amount of missing information. As a consequence, the interpretation of the findings was affected by the poor reporting in several studies.

### Limitations of this review

Our study has some limitations. The first is related to the scope of our search, which was conducted using only three databases, albeit the three key databases. Another limitation concerns the bias related to the publication of positive results. We did not contact experts in the field to identify unpublished studies (the so-called “gray literature”). Unfortunately, given the large heterogeneity of studies, it was not possible to obtain a summarizing measure of the results to quantify the effect of the interventions.

### Recommendations for future studies

Future studies should be reported according to the guidelines for controlled studies (e.g., CONSORT [46,47]) and non-randomized controlled studies (e.g., Strobe Statement [48]), thus avoiding the lack of crucial information for assessing the quality of these investigations. In addition, validated resilience scales should be used to assess the results. There is a need for improvement in the design of randomized observational trials, with an emphasis on randomized studies, as they are the gold standard for evaluating efficacy. Studies should also investigate factors which may mediate the effects of the intervention, yielding a certainty about the relative contribution of nonspecific factors in the outcome and identifying which specific program components can account for positive effects. The authors of the studies should state the characteristics of responders and non-responders or even of those who are adversely affected by the intervention.

Studies should also include follow-up data, and this should take place over a longer period after the intervention. Longitudinal samples of hazardous occupations such as police officers, soldiers and firefighters, are ideal for this type of study, as it is possible to evaluate the subjects on a baseline before they are exposed to hazardous situations. Moreover, resilience can be measured after the occurrence of dangerous situations.

## Conclusion

Most of the studies included reported some degree of improvement in resilience-like variables among those subjects exposed to resilience-promoting programs. This finding was more frequent among randomized controlled trials than in other study designs, which were included in this systematic review. Although comparing the number of studies with positive versus negative findings (“vote-counting”) has several limitations, poor operationalization of the constructs and the large heterogeneity in study designs and measurements prevented us from carrying out a meta-analysis. Nevertheless, it is worth noting that positive findings were more consistent among studies which used randomized designs, usually regarded as being less biased to evaluate interventions. These results, albeit fragile, point to the need to continue the investigation of the effectiveness of such intervention programs. It is imperative to design and conduct studies with a better methodology and better reporting (e.g., adherence to Prisma [49]) and to look for better evidence of the actual impact of resilience-promoting programs. Additional efforts should be made to determine the actual effects of the interventions, as this is crucial for the cost-effectiveness calculation of resilience-promoting strategies.

## Additional file

Additional file 1:
**Description of selected studies.**
